# Appendicular endometriosis: A case report and review of literature

**DOI:** 10.1016/j.ijscr.2019.07.046

**Published:** 2019-07-22

**Authors:** Rahul Gupta, Arvind K. Singh, Waad Farhat, Houssem Ammar, Mohamed Azzaza, Abdkader Mizouni, Sami lagha, Mehdi Ben Latifa, Amal Bouazzi, Ali Ben Ali

**Affiliations:** a Department of Gastrointestinal Sciences, Synergy Institute of Medical Sciences, Dehradun, India; b Department of Gastrointestinal Surgery, Sahloul Hospital, University of Medicine of Sousse, Tunisia

**Keywords:** Appendicular endometriosis, Case report, Endometriosis, Infertility, Pain, Right iliac fossa

## Abstract

**Introduction:**

Patients with appendicular endometriosis (AE) constitute <1% cases of all pelvic endometriosis cases. AE presents with pain in right iliac fossa and symptomatically mimics appendicitis and definitive diagnosis is possible only after histopathological examination of excised appendix.

**Presentation of case:**

In this case report, we present a rare case of appendicular endometriosis in a young woman suffering from pain in right iliac fossa and periumbilical region (on/off) for the past one year. She had a past history of infertility. Blood investigations were normal and CT scan of abdomen demonstrated findings suggestive of appendicitis. Laparoscopic appendicectomy was performed, which revealed 2 cm tumor-like mass (1.5 cm diameter) present at the tip of appendix involving the mesoappendix. There was no ascites, peritoneal or omental deposits or any signs of inﬂammation. Post-operative recovery was good. Dissected appendix specimen was sent for histopathological examination which confirmed endometriosis of appendix.

**Discussion:**

Gastrointestinal endometriosis (GE) accounts for 3–37% of all endometriosis cases, whereas AE is present in only ∼3% of all GE cases and constitutes <1% of all the endometriosis cases. AE frequently involves tip and body of the appendix. The layers of appendix most commonly affected are muscular and seromuscular (∼2/3rd cases), followed by the serosa (∼1/3rd cases).

**Conclusion:**

Thus, appendicular endometriosis is a rare entity and should be included in differential diagnosis in young women presenting with pain in right iliac fossa and having history of infertility.

## Highlights

• Appendicular endometriosis is a rare disease.
• Preoperative diagnosis is difficult.
• Laparoscopic appendicectomy is the treatment of choice.
• It should be included in differential diagnosis of lower abdominal pain in young women with history of infertility.

## 1 Introduction

Right iliac fossa pain remains one of the most common symptoms for surgical consultation [1]. In majority of the cases, acute appendicitis is contemplated as the initial diagnosis and it is unquestionably the most frequent cause. Appendectomy remains the most common emergency abdominal surgical procedure. However, it is still difficult to make a correct diagnosis and a recent study reports a negative appendicectomy rate of 9.2% [2]. In an American study, the total cost of negative appendectomy was found to be about 9–11 million USD per year [3]. Additionally, negative appendicectomy has been found to be associated higher median cost per admission, higher morbidity and longer hospital stay compared to patients with appendectomy for nonperforated appendicitis [4]. Hence, reduction in negative appendectomy rate is essential. Computed tomography of the abdomen has been found to be very useful investigation in detecting other causes of right iliac fossa pain and reduce the rate of negative appendectomy [5]. Other causes of pain in right iliac fossa includes right ureteric colic, torsion of an appendix epiploicae, amoebic typhilitis, inflammatory bowel disease, inflamed diverticulum of the caecum, ruptured ectopic gestation, acute cholecystitis, perforated duodenal ulcer and pelvic inflammatory disease (PID). Apart from these, rare causes could be an appendicular or caecal endometriosis [[6], [7], [8]].

The presence of endometrial glands and stroma outside the uterine cavity is known as endometriosis [9]. It remains an immensely prevalent disease and involves ∼10%, ∼70% and ∼50% of the women in their reproductive age, women with chronic pelvic pain, and those with infertility, respectively [10]. Appendicular endometriosis (AE) may remain asymptomatic or present as acute or chronic appendicitis, lower gastrointestinal (GI) bleeding, intestinal perforation, or intestinal obstruction as a result of intussusception [[9], [10], [11]]. Here, we report a case of AE which was preoperatively misdiagnosed as acute appendicitis but later detected on histopathology. This case has been reported in line with the SCARE criteria [12].

## 2 Case report

**Fig. 1:**
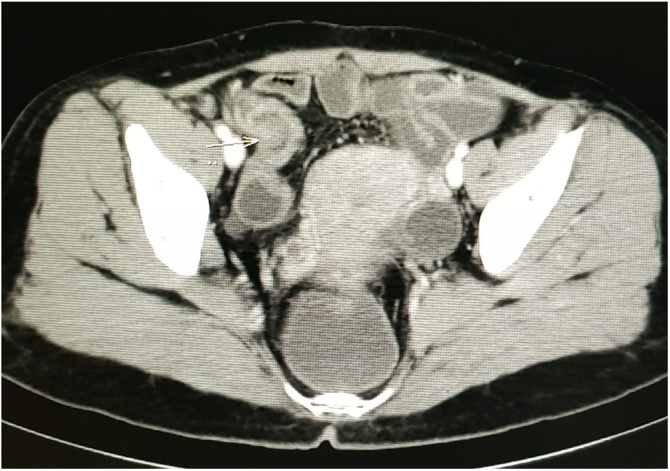
Contrast enhanced computed tomography of abdomen demonstrating a well-defined tubular structure (11 mm in luminal diameter) with lobulated tip (arrow) in relation to the cecum and terminal ileum.

A 35-year-old female came to the surgical out-patient department of our hospital with chief complaints of intermittent pain in the periumbilical region and right iliac fossa for one year. Pain was not associated with other complaints or menstruation. There was a history of infertility and no other significant past medical history. The physical examination was unremarkable. The routine blood investigations were normal. USG abdomen and pelvis revealed well-defined nodular lesion (1.5 × 1.3 × 1.6 cm) in right iliac fossa, and mild hypoechoic circumferential wall thickening in the terminal ileum and ileo-caecal junction (3–5 mm thick). Contrast enhanced computed tomography (CT) of abdomen revealed well-defined long tubular structure (11 mm in luminal diameter) with lobulated tip along the postero-medial wall of caecum, mild pericaecal stranding and thickened right lateral conal fascia – suggesting of acute appendicitis (Fig. 1). Additionally, multiple cysts were present in both the ovaries. In view of above findings, patient was planned for laparoscopic appendectomy after obtaining written informed consent.

**Fig. 2:**
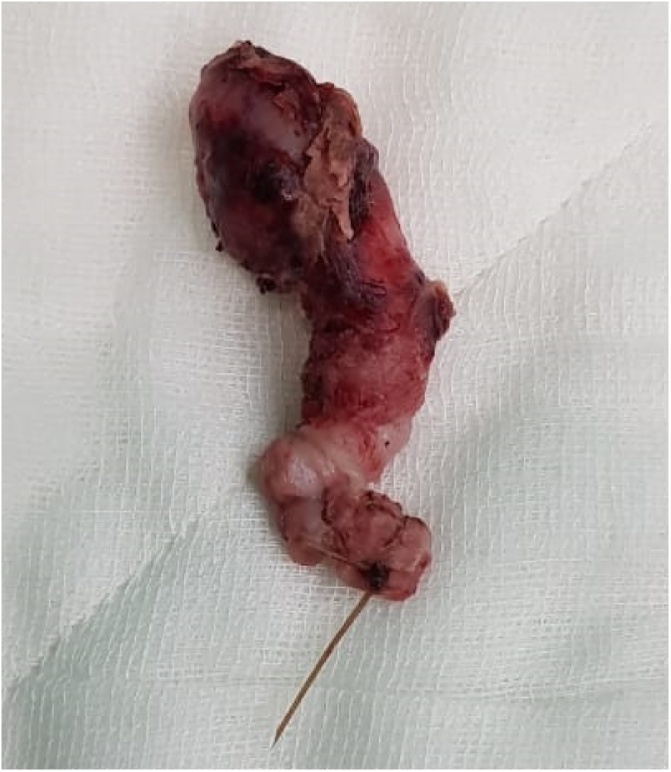
Excised appendix (measuring 2.5 × 2 × 5 cm) showing tumor-like mass at the tip (1.5 cm). Fig. 2 Appendicular E.

Intra-operatively, there was presence of a 2 cm tumor-like mass at the tip of appendix involving the mesoappendix, while base of the appendix was normal. There was no ascites, peritoneal or omental deposits or any signs of inﬂammation. The entire appendix measuring 2.5 × 2 × 5 cm was dissected and excised (Fig. 2). Caecum and terminal ileal loops were unremarkable. The operative time was 60 minutes with blood loss of 20 ml. The postoperative recovery was uneventful with the hospital stay of 3 days. Gross examination found a firm white nodule measuring 1.5 cm in diameter located at the tip of the appendix. Microscopic examination revealed the presence of endometrial glands, stroma with no signs of inflammation suggestive of appendicular endometriosis. Till the last follow up at 3 months, the patient was symptom-free.

## 3 Discussion

Endometriosis is described as an estrogen-dependent, chronic inflammatory disorder. Presence of viable endometrial tissue outside the uterus is the characteristic finding. It frequently involves the reproductive organs and peritoneum. It may also affect the GIT, omentum, mesentery and surgical scars, while the distant and rare sites includes the lungs, kidneys, nasal cavity and skin. Gastrointestinal endometriosis (GE) accounts for 3–37% of all endometriosis cases, whereas AE is present in only ∼3% of all GE cases and constitutes <1% of all the endometriosis cases [[13], [14], [15]].

The most frequent symptoms includes dysmenorrhea followed by chronic non-menstrual lower abdominal pain. Other symptoms are infertility, painful coitus, pain during defecation, painful bladder symptoms and dysuria, bowel symptoms (diarrhea, cramping, constipation), and ovarian mass [16]. Chronic pelvic pain in endometriosis is due to chronic exposure to systemic and local proinflammatory cytokines and growth factors which in turn results in peripheral sensitization distinguished by a hyperalgesia, myofascial pain, and central sensitization [17]. In women with endometriosis, finding of altered brain chemistry is strongly linked with pain intensity [18].

None of the available diagnostic procedures (i.e., transvaginal USG, MRI, Doppler ultrasonography, CA-125 levels) help in the preoperatively diagnosis of AE. A definitive diagnosis is possible only after histopathological examination of the excised appendix. Since laparoscopy provides a direct clue due to visualisation of features under magnification, it may be considered as a standard diagnostic method. However, when a young woman presents with complaints of chronic pelvic non-menstrual pain, and past history of infertility and pelvic endometriosis, diagnosis of AE must be suspected. Similarly, our patient was young and had a history of chronic lower abdominal pain and infertility, which helps in the diagnosis. Also, CT scan revealed findings suggestive of acute appendicitis.

AE frequently involves tip and body of the appendix. The layers of appendix most commonly affected are muscular and seromuscular (∼2/3rd cases), followed by the serosa (∼1/3rd cases). American Society for Reproductive Medicine has proposed a staging system, which categorises the endometriosis in to 4 stages: Stage I to IV i.e., minimal, mild, moderate and severe [19]. This staging system is frequently used to estimate the burden of the disease and assists in maintaining the homogeneity in both patient care and research. The characteristic histologic features are presence of endometrial glands, stroma, fibrosis, and hemosiderin-laden macrophages; chronic bleeding; and signs of inflammation. Similarly, in our case, the lesion was located at the tip of appendix and involved muscular and seromuscular layers. On microscopic examination, there was the presence endometrial glands, stroma, and but no signs of inflammation.

Surgical excision of affected tissue/organ is generally needed for the most efficient and exhaustive treatment of endometriosis. As per the recent guidelines, appendicectomy should be performed laparoscopically, unless contraindicated [20]. The management of endometriosis that has not deeply infiltrated can be done by ablation of the lesion with CO2 laser. In our patient, laparoscopic appendicectomy was performed. A recent study by Duffy and colleagues demonstrated that laparoscopic surgery when compared with diagnostic laparoscopy for endometriosis clearly results in reduced overall pain at 6 and 12 months. They are also reported that more women treated with laparoscopic ablation were pain free at 12 months, when compared with combination of diagnostic laparoscopy and GnRH agonist [21].

## 4 Conclusion

Appendicular endometriosis is a rare entity and almost always diagnosed after surgical excision of the appendix followed by histopathological examination. However, it should always be included in differential diagnosis when a young woman presents with complaint of chronic right iliac fossa pain mimicking appendicitis and has a history of infertility.

## Declaration of Competing Interest

The authors declare that they have no conflict of interest.

## Funding

This study did not receive any funding.

## Ethical approval

The study was approved by Ethics Committee of Hospital Sahloul Sousse.

## Consent

Written informed consent was obtained from the patient.

## Author contribution

Study concept or design – AS, HA.

Data collection – HA, WF, RG.

Data interpretation – MBL, WF, RG.

Literature review – WF, ABA, SL.

Drafting of the paper – HA, MA, SL.

Editing of the paper – AB, AS, AM.

## Registration of research studies

As this was a case report and not a clinical trial, this study does not require registration.

## Guarantor

Houssem Ammar.

## Provenance and peer review

Not commissioned, externally peer-reviewed.
